# Contrast echocardiography in myocardial infarction

**DOI:** 10.1590/S1679-45082013000300023

**Published:** 2013

**Authors:** Maria Luciana Zacarias Hannouche da Trindade, Marcelo Luiz Campos Vieira, Ana Clara Tude Rodrigues, Miguel José Francisco, Claudio Henrique Fischer, Samira Saady Morhy

**Affiliations:** 1 Hospital Israelita Albert Einstein, São Paulo, SP, Brazil.

**Keywords:** Myocardial infarction/ultrasonography, Echocardiography/methods

## Abstract

The contrast agents used in ultrasound are approved for several clinical situations. New echocardiographic techniques, such as harmonic imaging and power pulse inversion imaging, can improve the visualization of microbubbles. In this article we discuss the early development of contrast echocardiography, new technologies that help improve image acquisition and its practical role in the assessment of myocardial infarction.

## RESUMO

Os contrastes ecocardiográficos à base de microbolhas estão aprovados para várias situações da prática clínica. Novos *softwares*, como a imagem em segunda harmônica e o *power pulse inversion*, podem melhorar a visualização do contraste no exame ecocardiográfico. Neste artigo, discutem-se o início da prática da ecocardiografia com contraste, as novas tecnologias que ajudam na aquisição e melhora da imagem, e seu uso no infarto do miocárdio.

## INTRODUCTION

Contrast echocardiography is a technique that uses microbubble contrast agents administered via peripheral veins to improve the echocardiographic signal^(1)^. Technological advances and the use of contrast agents more stable in the circulation have positioned contrast echocardiography as a safe and effective technique for the assessment of myocardial perfusion^(2)^. The clinical applications of contrast echocardiography include the assessment of patients with suspected or known coronary artery disease; determination of the area at risk and efficacy of reperfusion therapies in patients with acute myocardial infarction; and assessment of myocardial viability after infarction (no-reflow phenomenon).

### Microbubble contrast agents for echocardiography

The use of agitated saline solution as an echocardiographic contrast agent to enhance blood within the heart was first described by Gramiak and Shah in 1968, based on the creation of a gas-blood interface^(3)^. Since these microbubbles were large, they would not cross the pulmonary barrier, so their use was limited to the opacification of the right cardiac chambers.

The sonication process developed by Feinstein et al. enabled the reduction of the microbubble size, thus permitting their passage through the pulmonary capillary circulation, and consequent opacification of the left cardiac chambers^(4)^. Because these microbubbles had a small diameter and were room-air filled, they persisted only for a short time in the systemic circulation, and dissolved in blood easily. Additionally, they underwent quick destruction when exposed to ultrasonic energy, which made it difficult for them to be applied in the study of the myocardial flow^(5)^.

The use of high molecular weight less diffusible gases resulted in the production of more stable contrast agents capable of remaining in the systemic circulation for a longer time^(6)^.

The first-generation contrast agents are composed of room-air-filled microbubbles. The composition of second-generation contrast agents, in turn, include high molecular weight gases; these agents are enclosed by a protein, lipid or chemical-polymer capsule, and show lower diffusivity and more stability, which permit their passage through the pulmonary capillary barrier. Thus, they can reach the left heart chambers and, consequently, the coronary microcirculation^(7)^.

### Imaging modalities

When hit by an ultrasound beam, the microbubbles vibrate and reflect waves with multiple sound frequencies from the fundamental frequency emitted by the ultrasound scanner, which are known as harmonics^(8)^. The development of new ultrasound systems capable of identifying second-harmonic signals permitted the formation of the “second-harmonic images”, with preferential detection of signals originating from the microbubbles. This imaging modality represented a great advance in the improvement of quality of two-dimensional images, because of the higher resolution of the image and definition of the endocardial borders provided by ventriculography obtained by the presence of microbubbles in the left ventricular (LV) chamber, with a consequent more adequate analysis of both the regional and global motion^(9)^.

**Figure 1:**
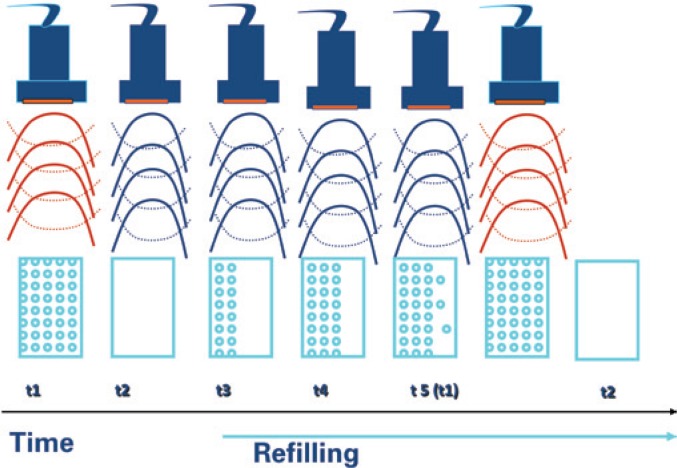
Diagram representing the intermittent ultrasound pulse emission technique. The first high-energy ultrasound pulse reaches the microbubbles in the microcirculation and destroys them. In this process, the microbubbles emit harmonics, which permit the generation of the echocardiographic image. After five heart beats, the microcirculation is already refilled, thus being able to receive a new ultrasound pulse for the image to be formed again

However, the use of high-energy ultrasound (high mechanical indexes), present during the performance of fundamental-image or even second-harmonic studies, cause the microbubbles to collapse. The fast destruction of microbubbles makes the assessment of the myocardial perfusion impossible when only second-harmonic imaging is used. A means to avoid this problem is to emit intermittent ultrasound pulses every two, three, four, or even ten heart beats. Thus, after a first pulse of high-energy ultrasound, there is complete microbubble destruction in a given ultrasonic field. Since the next ultrasound pulse is emitted only seconds after the first one, there is time for the microbubbles to refill all the microvascular bed, thus making the myocardial perfusion study possible, with acquisition of static images, without motion in real time (Figure 1).

### Real-time myocardial perfusion echocardiography

The study of myocardial perfusion by echocardiography implies the peripheral intravenous administration of microbubble contrast agents which, because of behaving similarly to the red blood cells, are excellent markers of myocardial flow, thus determining the integrity of the coronary microcirculation^(10)^.

**Figure 2:**
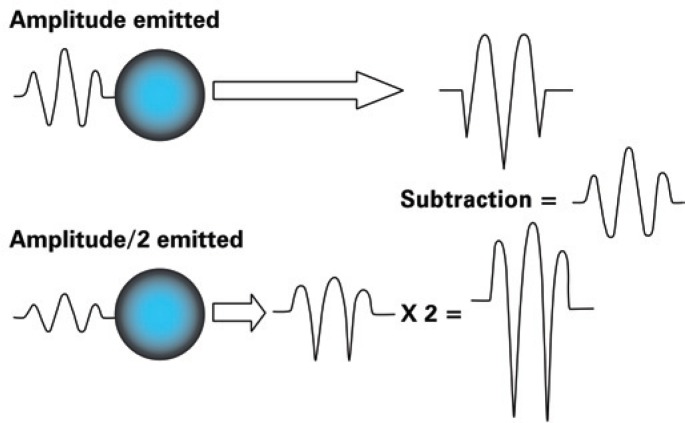
The alternating-amplitude pulse technique with the microbubbles

Recently, another imaging modality known as real-time myocardial perfusion echocardiography (RTMPE) was developed. It permits the concomitant analysis of myocardial reperfusion and segmental LV contraction. However, the signal originating in the microbubbles is relatively weak using this technique. With the purpose of overcoming this limitation, the alternating-amplitude pulse technique is used; this consists of the emission of two energy pulses in which a pulse is half the amplitude of the other. At reception, the pulse with half the amplitude is multiplied by two. These signals are subtracted, so that structures responding in a linear fashion (tissues) will show a reduction or absence of signal in the end, whereas structures responding in a non-linear fashion (microbubbles), will show a signal different from zero, thus being detected (Figure 2).

**Figure 3:**
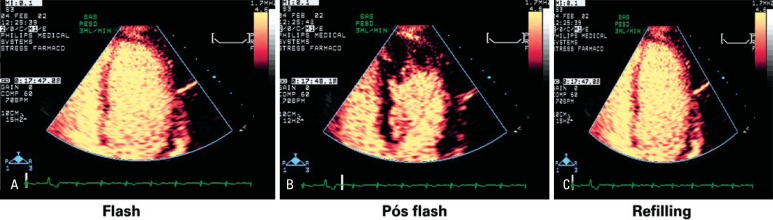
Echocardiographic images of real-time myocardial perfusion. (A) High ultrasound energy pulse (mechanical index - MI of 1.0). (B) Microbubble destruction in the myocardium. (C) Myocardial refilling by microbubbles

Since practically no microbubble destruction occurs, the myocardium becomes saturated with them, and their intensity remains constant. Then, there is another key component – the emission of two to five high ultrasound energy pulses, flash (Figure 3), for myocardial contrast destruction so that the rate of myocardial refilling with microbubbles can be documented and further measured, immediately after the high-energy pulses, without the need for alignment and image post-processing. This makes the method feasible and with a great potential for clinical applicability^(11)^.

Myocardial opacification has proven to be a promising path for the diagnosis and prognosis of patients with acute myocardial infarction (AMI), for having the potential of supplying qualitative and quantitative information on the regional and global LV microvascular flow^(12,13)^.

### Microvascular flow

The vascular compartment in the myocardium comprises the great arteries, arterioles, the capillary network and smaller intramyocardial veins. The coronary microcirculation is defined by being composed of vessels smaller than 200*μ*m in diameter. In this complex system, the total coronary flow corresponds to an average of 4% to 5% of the cardiac output and, in baseline conditions, 8% of the LV mass is comprised of the blood present in the microcirculation, 90% of which is within the capillaries, then known as myocardial blood volume^(14)^. The coronary blood flow velocity is directly related to the diameter of the vessels; it is approximately 40cm/s in epicardial arteries and 1mm/s or less in the capillaries of humans^(15)^. The microcirculation is not only a passive channel through which blood is transported in the myocardium, but rather an active site of blood flow control with a complex regulation which depends on metabolic and myogenic mechanisms. For the capillary exchange to be preserved, several physiological mechanisms keep the capillary hydrostatic pressure constant in the myocardium at approximately 30mmHg, with pre and post-capillary pressures of approximately 45 and 15mmHg, respectively^(16)^. The coronary arterioles act as resistance vessels. They have smooth muscle and show immediate myogenic response, and that is why the arteriolar resistance can change from second to second in order to keep the pre-capillary pressure constant. On the other hand, the coronary venules present a poor myogenic response and control the local resistance by changing the rheological properties of the blood. Capillaries are very small and offer high resistance. However, since they are arranged in parallel, the total capillary resistance decreases with the increase in the number of capillaries. At rest, approximately 60% of the total myocardial vascular resistance is offered by the arterioles, 25% by the capillaries, and 15% by the venules^(17)^. By means of retrograde stimuli, usually measured by the increase in oxygen consumption, higher or lower capillary recruitment can occur. In baseline conditions, approximately half of them is not working^(18)^.

### Myocardial flow quantification

Recently, it has been demonstrated that ultrasound-induced microbublle destruction can be used for the measurement of the myocardial blood flow during continuous infusion of a contrast agent. The relative agent concentration in different myocardial beds represents the capillary density or the sum of its crosssectional area. Microbubbles may be destroyed by some high-energy ultrasound pulses and, from then on, their rate of reappearance in the myocardium may be measured, and represents the mean microbubble velocity in the microcirculation. This is possible because, after being destroyed, the time available for another microbubble inflow to cover any distance within the ultrasound beam is determined by the increase in the acoustic intensity (AI) in a given ultrasound pulse interval (the interval between the formation of two images). On the other hand, the microbubble concentration in the microcirculation is directly proportional to AI, and reflects the microvascular blood volume. Thus, for any pulse interval, the AI within the beam will be proportional to the distance covered by the microbubbles within the beam. Since the microbubble velocity is constant in the microcirculation, the AI will be greater as the time of exposure to lowenergy ultrasound increases, up to the moment when capillary saturation within the ultrasound beam is reached. Therefore, the AI, at this moment, will remain constant, thus reflecting the relative concentration of microbubbles in the tissues.

When the microbubbles are continuously administered intravenously, at a constant infusion rate and concentration, a steady state is achieved, in which the microbubble concentration in the blood is equal to that in the myocardium. Thus, considering that the microbubble concentration within the myocardium is proportional to the blood volume fraction, and knowing the blood flow velocity, it is possible to estimate the myocardial blood flow^(19)^.

There are three models of myocardial blood flow quantification using contrast echocardiography. The first is the visual analysis, which is semiquantitative, subjective, limited to the observer's experience, and based on the determination of perfusion scores that range from normal perfusion to absent perfusion. This commonly assesses only the microvascular blood volume component and, due to a limitation of the human's eye perception, valuable information on the blood flow velocity is missed. The second is myocardial blood volume quantification (represented by the letter A), and of the myocardial blood flow velocity (represented by the Greek letter ß) in regions of interest (ROI) in the myocardium, using a specific computer program. The third model consists of parametric imaging, a novel method of automated quantification in which the myocardial blood flow is codified in colors all over the myocardium, according to the degree of perfusion. Color grades are linearly applied based on mean values, with cut-off points fixed in the mean values (green), values between two thirds and one third of the mean values (yellow), and values below one third of the mean value (red), corresponding to normal, decreased, and significantly decreased perfusion, respectively.

### Assessment of the collateral circulation after acute myocardial infarction and the no-reflow phenomenon

Sabia et al. studied 43 patients who had had an inferior myocardial infarction two days to five weeks earlier and occluded right coronary artery using direct contrast injection in the left coronary artery. They demonstrated that, after the intracoronary injection, there was opacification of the whole myocardium in 32 patients, thus suggesting collateral circulation for the infarct area. On the other hand, coronary angiography did not show abundant collateral circulation, thus demonstrating that contrast echocardiography is more accurate in the assessment of collateral vessels, since angiography only defines vessels greater than 100*μ*m. In this study, 78% of patients with adequate collateral circulation for the infarct bed on contrast echocardiography showed improvement in the regional systolic function 30 days after flow was reestablished. Of these, only 17 patients showed collateral circulation on angiography. These data indicate that, in addition to the duration of coronary occlusion, the extent of residual myocardial perfusion is also determinant of the infarct size^(20)^.

Acute coronary occlusion leads to cell necrosis and consequent permanent functional myocardial damage. Kloner et al. assessed the event time in an experimental model and demonstrated that after 40 minutes of ischemia for coronary occlusion, a variable amount of myocytes was necrotic, whereas the microvascular network was still intact. Ninety minutes or longer after coronary occlusion, a large percentage of myocardial cells was damaged, and the microvasculature showed loss of its anatomical integrity^(21)^. The moment the coronary was opened, reflow was found only in the areas with an anatomically preserved microvasculature, whereas no reflow was observed in the areas with a damaged microvasculature. This failure to achieve reperfusion following prolonged ischemia, first postulated by Krug et al. and later demonstrated by Kloner et al., is called the no-reflow phenomenon^(21,22)^.

In an experimental model (90 minutes of coronary occlusion followed by reperfusion), Jeremy et al. showed that, in the first 4 hours after the opening of the coronary artery, the region without microvascular perfusion became progressively larger in the area related to the infarct, and this occurred because of microvascular occlusion by neutrophils in the postischemic myocardium^(23)^. In a similar experimental model, Ambrosio et al. found a progressive loss in the intramyocardial reflow, with a late no-reflow area (3 to 5 hours after reperfusion) almost three times larger than the no-reflow area after early reperfusion (2 minutes after reperfusion)^(24)^.

Using microbubble contrast echocardiography via intracoronary injection, Ito et al. studied the no-reflow phenomenon in patients with anterior AMI who had an early recanalization (within 6 hours from the onset of chest pain) and demonstrated that the angiographic success of reflow (TIMI grade 3 flow) did not necessarily indicate an adequate myocardial perfusion^(25)^.

In another study of patients with anterior-wall AMI undergoing primary angioplasty, Ito et al. used intracoronary injection of microbubble contrast and demonstrated that, if more than 25% of the area at risk remained with a perfusion defect after recanalization, incomplete reperfusion would occur, thus determining what they defined as no-reflow. In the 30-day follow-up, the LV ejection fraction improved only in the group with TIMI grade 3 flow and absence of no-reflow^(26)^. Thus, the no-reflow phenomenon is an expression of microvascular damage and is associated with more extensive myocardial necrosis and worse regional and global contractile function.

Caldas et al. used echocardiography with intermittent imaging and contrast administered via a peripheral vein to study 31 patients within the first 48 hours of a primary anterior-wall AMI successfully treated with fibrinolytic drugs or percutaneous coronary intervention. The LV myocardial perfusion score was demonstrated to be an independent predictor of left ventricular remodeling and was proportional to the segmental contractility index score in the 6-month follow-up. The presence of the no-reflow phenomenon had a greater impact on the left ventricular remodeling than in the myocardial segments with reduced perfusion (low-reflow). After 6 months, patients with more than two myocardial segments without opacification after contrast showed functional recovery or contractile reserve in only 28% of the myocardial segments supplied by the anterior descending coronary artery. On the other hand, patients with two or less segments without opacification showed, in 6 months, recovery of the contractile function at rest or contractile reserve in 70% of the segments supplied by the anterior descending coronary artery^(27)^.

Sbano et al. studied 50 patients with AMI treated with fibrinolytic therapy and demonstrated that the group that progressed with recovery of the left ventricular function within 6 months showed perfusion in 65.8% of the infarct-related segments on contrast echocardiography with intermittent imaging performed in the first week after the event. The group presenting perfusion in only 25.5% of the infarct-related segments, in turn, progressed with no improvement of the left ventricular function. All myocardial segments without perfusion on contrast echocardiography showed no recovery of the contractile function within a period of 6 months^(28)^.

Trindade et al. observed an excellent correlation between real-time myocardial perfusion echocardiography, specially the parametric imaging, and magnetic resonance imaging, as regards the measurements of the infarct size and its percentage, as well as in the determination of its transmural extent in 30 patients hospitalized for primary AMI treated with fibrinolytic drugs or primary percutaneous intervention within up to 12 hours after the event^(29)^.

In summary, contrast echocardiography has a good safety and efficacy in the assessment of coronary artery disease^(30)^.
